# Butane-1,4-diammonium bis­(pyridine-2,6-dicarboxyl­ato-κ^3^ *O* ^2^,*N*,*O* ^6^)cadmate(II) dihydrate

**DOI:** 10.1107/S1600536808029395

**Published:** 2008-09-20

**Authors:** Masoumeh Tabatabaee, Hossein Aghabozorg, Roghaieh Nasrolahzadeh, Leila Roshan, Najmeh Firoozi

**Affiliations:** a Department of Chemistry, Islamic Azad University, Yazd Branch, Yazd, Iran; b Faculty of Chemistry, Tarbiat Moallem University, 49 Mofateh Avenue, Tehran, Iran

## Abstract

In the title compound, (C_4_H_14_N_2_)[Cd(C_7_H_3_NO_4_)_2_]·2H_2_O, the Cd^II^ ion is coordinated by four O atoms [Cd—O = 2.2399 (17)–2.2493 (17) Å] and two N atoms [Cd—N = 2.3113 (15) and 2.3917 (15) Å] from two tridentate pyridine-2,6-dicarboxyl­ato ligands in a distorted octa­hedral geometry. The uncoordinated water mol­ecules are involved in O—H⋯O and N—H⋯O hydrogen bonds, which contribute to the formation of a three-dimensional supra­molecular structure, along with π–π stacking inter­actions [centroid–centroid distances of 3.5313 (13) and 3.6028 (11) Å between the pyridine rings of neighbouring dianions].

## Related literature

For related literature, see: Aghabozorg, Firoozi *et al.* (2008 ▶); Aghabozorg, Manteghi *et al.* (2008 ▶); Odoko *et al.* (2002 ▶).
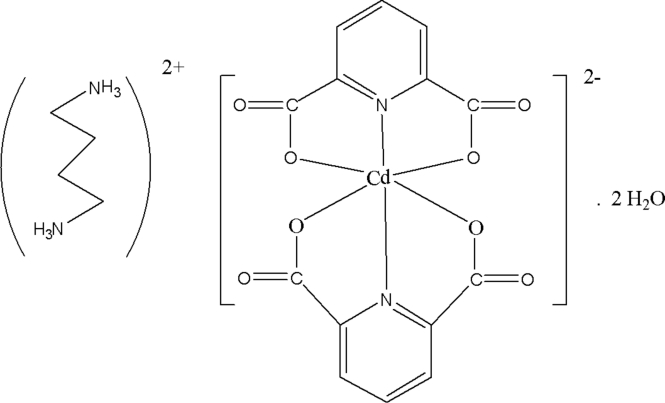


## Experimental

### 

#### Crystal data

(C_4_H_14_N_2_)[Cd(C_7_H_3_NO_4_)_2_]·2H_2_O
*M* *_r_* = 568.81
Monoclinic, 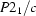

*a* = 11.0357 (4) Å
*b* = 28.7181 (10) Å
*c* = 7.1116 (3) Å
β = 108.544 (1)°
*V* = 2136.82 (14) Å^3^
*Z* = 4
Mo *K*α radiation
μ = 1.09 mm^−1^
*T* = 100 (2) K
0.28 × 0.07 × 0.05 mm

#### Data collection

Bruker SMART APEXII CCD area detector diffractometer
Absorption correction: multi-scan (*APEX2*; Bruker, 2005 ▶) *T*_min_ = 0.802, *T*_max_ = 0.944
25988 measured reflections
5676 independent reflections
4655 reflections with *I* > 2σ(*I*)
*R*_int_ = 0.044

#### Refinement

*R*[*F* ^2^ > 2σ(*F* ^2^)] = 0.027
*wR*(*F* ^2^) = 0.066
*S* = 0.99
5676 reflections
298 parameters
H-atom parameters constrained
Δρ_max_ = 0.59 e Å^−3^
Δρ_min_ = −0.71 e Å^−3^

### 

Data collection: *APEX2* (Bruker, 2005 ▶); cell refinement: *APEX2*; data reduction: *APEX2*; program(s) used to solve structure: *SHELXTL* (Sheldrick, 2008 ▶); program(s) used to refine structure: *SHELXTL*; molecular graphics: *SHELXTL*; software used to prepare material for publication: *SHELXTL*.

## Supplementary Material

Crystal structure: contains datablocks I, global. DOI: 10.1107/S1600536808029395/cv2442sup1.cif

Structure factors: contains datablocks I. DOI: 10.1107/S1600536808029395/cv2442Isup2.hkl

Additional supplementary materials:  crystallographic information; 3D view; checkCIF report

## Figures and Tables

**Table 1:** Hydrogen-bond geometry (Å, °)

*D*—H⋯*A*	*D*—H	H⋯*A*	*D*⋯*A*	*D*—H⋯*A*
N3—H3N*A*⋯O2*W*	0.89	1.90	2.782 (2)	169
N3—H3N*B*⋯O7^i^	0.91	1.93	2.823 (2)	166
N3—H3N*C*⋯O4^ii^	0.90	1.93	2.784 (2)	159
N4—H4N*A*⋯O8^ii^	0.91	1.99	2.821 (3)	152
N4—H4N*B*⋯O6^iii^	0.94	1.93	2.865 (2)	176
N4—H4N*C*⋯O1*W*^iv^	0.86	1.94	2.797 (2)	175
O1*W*—H1*WA*⋯O5^iv^	0.76	1.92	2.678 (2)	173
O1*W*—H1*WB*⋯O2	0.79	1.87	2.653 (2)	172
O2*W*—H2*WA*⋯O4^v^	0.82	1.99	2.809 (2)	175
O2*W*—H2*WB*⋯O1*W*	0.82	1.97	2.781 (2)	170

Symmetry codes: (i) 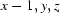; (ii) 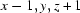; (iii) 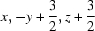; (iv) 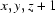; (v) 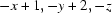.

**Fig. 1.**
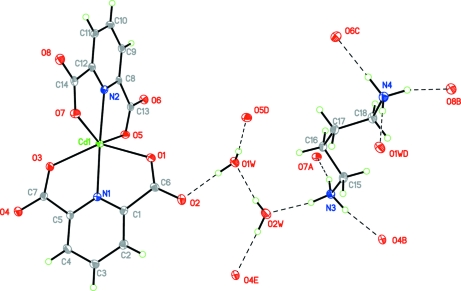
A portion of the crystal structure of I showing the atomic numbering, hydrogen bonds (dashed lines) and 50% displacement ellipsoids [symmetry codes: (A) x - 1,y,z; (B) x - 1,y,z + 1; C) x,-y + 3/2,z + 3/2; (D) x,y,z + 1; (E) -x + 1,-y + 2,-z].

**Fig. 2.**
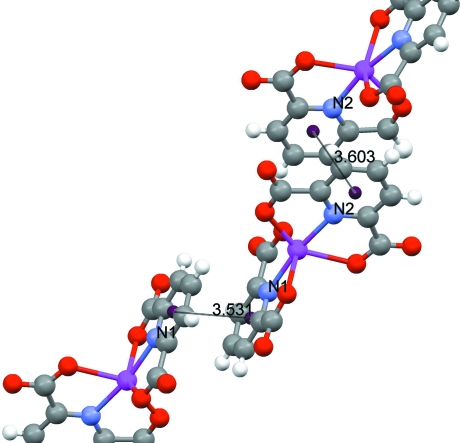
A portion of the crystal packing showing the π-π stacking interactions between the aromatic rings of the pydc2– dianions as dashed lines.

## supplementary crystallographic information

### Comment

Our research group has recently focused on a one-pot synthesis of water soluble self-assembly systems that can function as suitable ligands in the synthesis of metal complexes. In connection with this research area, pyridine2,6-dicarboxylic acid (pydcH_2_) has been selected as a proton donor and different amines as acceptor agents, and several metal complexes of these systems have been synthesized and their X-ray crystal structures reported (Aghabozorg, Firoozi  *et al.*, 2008; Aghabozorg, Manteghi  *et al.*, 2008). The major intermolecular interactions that are required in the preparation of supramolecular metal complexes have been present in most of these complexes. In order to develop novel systems, we report here a new complex of Cd^II^ with butane-1,4-diammonium pyridine-2,6-dicarboxylato as a proton transfer compound.

The title compound, I, consists of [Cd(pydc)_2_]^2-^ anion (pydcH_2_= pyridine-2,6-dicarboxylic acid), (bdaH_2_)^2+^ cation (bda=butane-1,4-diamine) and two uncoordinated water molecules (Fig. 1). Cd^II^ atom is six-coordinated by the four O and two N atoms from two (pydc)^2-^ ligands. The coordinating bond lengths (Cd—N 2.2399 (17) and 2.2493 (17) Å, and Cd—O 2.3113 (15), 2.3370 (15),  2.3433 (14) and 2.3917 (15) Å) lie in the normal ranges corresponding to those in realated complexes of Cd^II^ containing pyridine-2,6-dicarboxylate as a ligand (*Odoko *et al.*, 2002*). The bond lengths and bond angles around of metal center indicate that the geometric arrangement of six donor atoms around the Cd^II ^atom is distorted octahedral.

The N1—Cd1—N2 angle (170.36 (6)°) deviates slightly from linearity. The O1–Cd1–O5 and O3–Cd1–O7 bond angles and O1–Cd1–O5—C13 and O1–Cd1–O7—C14 torsion angles are 89.41 (6)°, 94.81 (5)°, -101.88 (14)° and 105.15 (14)°, respectively, indicating that two dianionic(pydc)^2– ^fragments are almost perpendicular to each other.The bond angles O1–Cd1–O3 [141.50 (5)^°^] and O5–Cd1–O7 [141.06 (5)^°^] indicate that the four carboxylate groups of the two dianions are oriented in a flattened tetrahedral arrangement around the Cd^II^atom.

In the crystal, there are  O—H···O and N—H···O hydrogen bonding interactions between the cations, anions and uncoordinated water molecules (Table 1). The water molecules acts also as bridging agents and link the cations to anions *via* hydrogen bonds (Fig. 1) and therefore, the spaces between two layers of [Cd(pydc)_2_]^2–^ anions are filled with (badH_2_)^2+^ cations and water molecules. There are also π-π stacking interactions (Fig. 2) between the aromatic rings of the coordinated (pydc)^2-^ anions proved by short distances  *Cg*1···*Cg*1(1-*x*,2-y,-*z*) of 3.5313 (13) Å (Cg1 is a centroid of N1/C2—C5), and *Cg*2···*Cg*2(*x*,3/2-y,1/2+*z*) of 3.6028 (11) Å (*Cg*2 is a centroid of N2/C8—C12). Ion pairing, hydrogen bonding and π–π stacking interactions stabilize the crystal packing.

### Experimental

A mixture of an aqueous solution (30 ml) of the proton transfer compound (bdaH_2_)(pydc) (100 mg, 0.4 mmol) and cadmium(II) nitrate Cd(NO_3_)_2_. 4H_2_O, (60 mg, 0.2 mmol) were stirred at 0°C. Colorless crystals of the title compound were obtained after 2 months at room temperature.

### Refinement

C-bound H atoms were placed in calculated positions. Positions of N- and O-bound  H atoms were found on a difference Fourier map. All hydrogen atoms were refined in riding model approximation, with *U*_iso_(H) equal to 1.2 *U*_eq_(C) and 1.5 *U*_eq_(N, O).

### Crystal data

**Table:**

(C_4_H_14_N_2_)[Cd(C_7_H_3_NO_4_)_2_]·2H_2_O	*F*(000) = 1152
*M**_r_* = 568.81	*D*_x_ = 1.768 Mg m^−^^3^
Monoclinic, *P*2_1_/*c*	Mo *K*α radiation, λ = 0.71073 Å
Hall symbol: -P 2ybc	Cell parameters from 6372 reflections
*a* = 11.0357 (4) Å	θ = 2.8–32.1°
*b* = 28.7181 (10) Å	µ = 1.09 mm^−^^1^
*c* = 7.1116 (3) Å	*T* = 100 K
β = 108.544 (1)°	Prism, colourless
*V* = 2136.82 (14) Å^3^	0.28 × 0.07 × 0.05 mm
*Z* = 4	

### Data collection

**Table:**

Bruker SMART APEX II CCD area detector diffractometer	5676 independent reflections
Radiation source: fine-focus sealed tube	4655 reflections with *I* > 2σ(*I*)
graphite	*R*_int_ = 0.044
phi and ω scans	θ_max_ = 29.0°, θ_min_ = 2.0°
Absorption correction: multi-scan (APEX2; Bruker, 2005)	*h* = −15→15
*T*_min_ = 0.802, *T*_max_ = 0.944	*k* = −39→39
25988 measured reflections	*l* = −9→9

### Refinement

**Table:**

Refinement on *F*^2^	Primary atom site location: structure-invariant direct methods
Least-squares matrix: full	Secondary atom site location: difference Fourier map
*R*[*F*^2^ > 2σ(*F*^2^)] = 0.027	Hydrogen site location: mixed
*wR*(*F*^2^) = 0.066	H-atom parameters constrained
*S* = 1.00	*w* = 1/[σ^2^(*F*_o_^2^) + (0.0369*P*)^2^ + 0.2951*P*] where *P* = (*F*_o_^2^ + 2*F*_c_^2^)/3
5676 reflections	(Δ/σ)_max_ = 0.001
298 parameters	Δρ_max_ = 0.59 e Å^−^^3^
0 restraints	Δρ_min_ = −0.71 e Å^−^^3^

### Special details

**Table:**

Geometry. All e.s.d.'s (except the e.s.d. in the dihedral angle between two l.s. planes) are estimated using the full covariance matrix. The cell e.s.d.'s are taken into account individually in the estimation of e.s.d.'s in distances, angles and torsion angles; correlations between e.s.d.'s in cell parameters are only used when they are defined by crystal symmetry. An approximate (isotropic) treatment of cell e.s.d.'s is used for estimating e.s.d.'s involving l.s. planes.
Refinement. Refinement of *F*^2^ against ALL reflections. The weighted *R*-factor *wR* and goodness of fit *S* are based on *F*^2^, conventional *R*-factors *R* are based on *F*, with *F* set to zero for negative *F*^2^. The threshold expression of *F*^2^ > σ(*F*^2^) is used only for calculating *R*-factors(gt) *etc*. and is not relevant to the choice of reflections for refinement. *R*-factors based on *F*^2^ are statistically about twice as large as those based on *F*, and *R*- factors based on ALL data will be even larger.

### Fractional atomic coordinates and isotropic or equivalent isotropic displacement parameters (Å^2^)

**Table:**

	*x*	*y*	*z*	*U*_iso_*/*U*_eq_	
Cd1	0.653305 (14)	0.865460 (5)	0.02554 (2)	0.01027 (5)	
O1	0.54740 (15)	0.88396 (5)	0.2485 (2)	0.0163 (3)	
O2	0.49107 (15)	0.94185 (5)	0.4137 (2)	0.0177 (3)	
O3	0.74579 (14)	0.90014 (5)	−0.1942 (2)	0.0133 (3)	
O4	0.80812 (14)	0.96709 (5)	−0.2947 (2)	0.0148 (3)	
O5	0.46311 (14)	0.83512 (5)	−0.1895 (2)	0.0130 (3)	
O6	0.35049 (14)	0.77036 (5)	−0.2989 (2)	0.0134 (3)	
O7	0.85227 (14)	0.84106 (5)	0.2568 (2)	0.0135 (3)	
O8	0.97512 (14)	0.77840 (5)	0.3736 (2)	0.0140 (3)	
N1	0.66045 (16)	0.94278 (6)	0.0692 (2)	0.0107 (3)	
N2	0.66196 (16)	0.78722 (6)	0.0372 (2)	0.0094 (3)	
C1	0.61249 (19)	0.96095 (7)	0.2039 (3)	0.0106 (4)	
C2	0.6181 (2)	1.00835 (7)	0.2422 (3)	0.0130 (4)	
H2A	0.5863	1.0207	0.3381	0.016*	
C3	0.6726 (2)	1.03710 (7)	0.1332 (3)	0.0144 (4)	
H3A	0.6780	1.0690	0.1560	0.017*	
C4	0.7193 (2)	1.01781 (7)	−0.0105 (3)	0.0137 (4)	
H4A	0.7546	1.0366	−0.0861	0.016*	
C5	0.71190 (19)	0.96990 (7)	−0.0379 (3)	0.0106 (4)	
C6	0.5459 (2)	0.92595 (7)	0.2994 (3)	0.0119 (4)	
C7	0.75859 (19)	0.94381 (7)	−0.1888 (3)	0.0108 (4)	
C8	0.56098 (19)	0.76257 (7)	−0.0730 (3)	0.0096 (4)	
C9	0.5627 (2)	0.71438 (7)	−0.0708 (3)	0.0119 (4)	
H9A	0.4929	0.6975	−0.1489	0.014*	
C10	0.67082 (19)	0.69158 (7)	0.0503 (3)	0.0120 (4)	
H10A	0.6738	0.6592	0.0553	0.014*	
C11	0.7742 (2)	0.71772 (7)	0.1635 (3)	0.0117 (4)	
H11A	0.8473	0.7032	0.2451	0.014*	
C12	0.76656 (19)	0.76590 (7)	0.1527 (3)	0.0100 (4)	
C13	0.44812 (19)	0.79128 (7)	−0.1984 (3)	0.0106 (4)	
C14	0.87469 (19)	0.79762 (7)	0.2713 (3)	0.0108 (4)	
N3	−0.04463 (17)	0.90758 (6)	0.5575 (2)	0.0125 (3)	
H3NA	0.0079	0.9221	0.5045	0.019*	
H3NB	−0.0818	0.8834	0.4763	0.019*	
H3NC	−0.1029	0.9278	0.5727	0.019*	
N4	0.20003 (17)	0.80268 (6)	1.2890 (3)	0.0129 (4)	
H4NA	0.1477	0.7916	1.3553	0.019*	
H4NB	0.2523	0.7791	1.2663	0.019*	
H4NC	0.2430	0.8260	1.3538	0.019*	
C15	0.0263 (2)	0.89086 (8)	0.7619 (3)	0.0140 (4)	
H15B	−0.0345	0.8793	0.8238	0.017*	
H15C	0.0716	0.9169	0.8404	0.017*	
C16	0.1212 (2)	0.85258 (8)	0.7631 (3)	0.0131 (4)	
H16B	0.1777	0.8631	0.6915	0.016*	
H16C	0.0753	0.8255	0.6947	0.016*	
C17	0.2010 (2)	0.83878 (8)	0.9735 (3)	0.0137 (4)	
H17A	0.2623	0.8152	0.9671	0.016*	
H17B	0.2482	0.8657	1.0409	0.016*	
C18	0.11864 (19)	0.82013 (8)	1.0923 (3)	0.0130 (4)	
H18A	0.0632	0.8447	1.1110	0.016*	
H18B	0.0652	0.7950	1.0195	0.016*	
O1W	0.32860 (14)	0.88159 (5)	0.4844 (2)	0.0142 (3)	
H1WA	0.3663	0.8704	0.5821	0.021*	
H1WB	0.3741	0.8990	0.4520	0.021*	
O2W	0.12203 (15)	0.94242 (6)	0.3694 (2)	0.0211 (4)	
H2WA	0.1440	0.9692	0.3542	0.032*	
H2WB	0.1883	0.9273	0.4046	0.032*	

### Atomic displacement parameters (Å^2^)

**Table:**

	*U*^11^	*U*^22^	*U*^33^	*U*^12^	*U*^13^	*U*^23^
Cd1	0.01105 (8)	0.00794 (7)	0.01223 (8)	−0.00025 (6)	0.00430 (5)	−0.00009 (6)
O1	0.0229 (8)	0.0108 (7)	0.0201 (8)	0.0005 (6)	0.0135 (7)	0.0013 (6)
O2	0.0222 (8)	0.0159 (8)	0.0205 (8)	−0.0007 (6)	0.0148 (7)	0.0002 (6)
O3	0.0156 (7)	0.0113 (7)	0.0159 (7)	−0.0016 (6)	0.0091 (6)	−0.0021 (6)
O4	0.0179 (8)	0.0141 (8)	0.0159 (7)	−0.0007 (6)	0.0102 (6)	0.0015 (6)
O5	0.0121 (7)	0.0112 (7)	0.0144 (7)	0.0005 (6)	0.0025 (6)	0.0019 (6)
O6	0.0105 (7)	0.0154 (8)	0.0126 (7)	−0.0021 (6)	0.0012 (6)	−0.0001 (6)
O7	0.0126 (7)	0.0115 (7)	0.0144 (7)	−0.0007 (6)	0.0015 (6)	−0.0006 (6)
O8	0.0094 (7)	0.0175 (8)	0.0138 (7)	0.0000 (6)	0.0017 (6)	0.0019 (6)
N1	0.0109 (8)	0.0102 (8)	0.0118 (8)	0.0001 (6)	0.0048 (7)	0.0001 (6)
N2	0.0100 (8)	0.0106 (8)	0.0079 (8)	0.0002 (6)	0.0033 (6)	−0.0012 (6)
C1	0.0101 (9)	0.0128 (10)	0.0089 (9)	0.0011 (8)	0.0030 (7)	0.0005 (8)
C2	0.0135 (10)	0.0135 (10)	0.0126 (10)	−0.0012 (8)	0.0051 (8)	−0.0041 (8)
C3	0.0179 (11)	0.0110 (10)	0.0161 (10)	−0.0017 (8)	0.0080 (8)	−0.0017 (8)
C4	0.0141 (10)	0.0130 (10)	0.0152 (10)	−0.0025 (8)	0.0062 (8)	0.0007 (8)
C5	0.0095 (9)	0.0122 (10)	0.0108 (9)	0.0000 (7)	0.0042 (7)	0.0006 (7)
C6	0.0134 (10)	0.0120 (10)	0.0100 (9)	0.0006 (8)	0.0034 (8)	0.0029 (8)
C7	0.0094 (9)	0.0116 (10)	0.0115 (9)	−0.0002 (7)	0.0035 (8)	−0.0013 (7)
C8	0.0099 (10)	0.0123 (10)	0.0082 (9)	−0.0004 (7)	0.0050 (7)	0.0016 (7)
C9	0.0138 (10)	0.0121 (10)	0.0110 (9)	−0.0015 (8)	0.0059 (8)	−0.0005 (8)
C10	0.0160 (11)	0.0092 (9)	0.0122 (10)	0.0005 (8)	0.0066 (8)	0.0002 (7)
C11	0.0118 (10)	0.0133 (10)	0.0109 (9)	0.0036 (8)	0.0046 (8)	0.0030 (8)
C12	0.0102 (9)	0.0129 (10)	0.0078 (9)	0.0002 (7)	0.0040 (7)	0.0006 (7)
C13	0.0104 (10)	0.0134 (10)	0.0091 (9)	0.0009 (8)	0.0045 (8)	0.0013 (7)
C14	0.0111 (10)	0.0135 (10)	0.0091 (9)	−0.0017 (8)	0.0050 (8)	−0.0005 (7)
N3	0.0126 (8)	0.0126 (9)	0.0126 (8)	−0.0006 (7)	0.0046 (7)	0.0000 (7)
N4	0.0126 (9)	0.0141 (9)	0.0121 (8)	0.0005 (7)	0.0042 (7)	0.0002 (7)
C15	0.0133 (10)	0.0181 (11)	0.0108 (10)	−0.0004 (8)	0.0042 (8)	−0.0019 (8)
C16	0.0122 (10)	0.0155 (10)	0.0126 (10)	0.0012 (8)	0.0054 (8)	0.0010 (8)
C17	0.0121 (10)	0.0159 (11)	0.0143 (10)	0.0014 (8)	0.0058 (8)	0.0010 (8)
C18	0.0093 (10)	0.0171 (11)	0.0119 (10)	0.0001 (8)	0.0023 (8)	0.0018 (8)
O1W	0.0137 (8)	0.0153 (7)	0.0135 (7)	−0.0005 (6)	0.0044 (6)	0.0037 (6)
O2W	0.0187 (8)	0.0174 (8)	0.0304 (9)	0.0010 (6)	0.0123 (7)	0.0074 (7)

### Geometric parameters (Å, °)

**Table:**

Cd1—N1	2.2399 (17)	C9—H9A	0.9300
Cd1—N2	2.2493 (17)	C10—C11	1.388 (3)
Cd1—O1	2.3113 (15)	C10—H10A	0.9300
Cd1—O5	2.3370 (15)	C11—C12	1.387 (3)
Cd1—O3	2.3433 (14)	C11—H11A	0.9300
Cd1—O7	2.3917 (15)	C12—C14	1.525 (3)
O1—C6	1.261 (3)	N3—C15	1.494 (3)
O2—C6	1.244 (3)	N3—H3NA	0.8893
O3—C7	1.262 (2)	N3—H3NB	0.9137
O4—C7	1.255 (2)	N3—H3NC	0.8970
O5—C13	1.269 (2)	N4—C18	1.488 (3)
O6—C13	1.242 (2)	N4—H4NA	0.9110
O7—C14	1.269 (2)	N4—H4NB	0.9372
O8—C14	1.245 (2)	N4—H4NC	0.8628
N1—C5	1.336 (3)	C15—C16	1.517 (3)
N1—C1	1.339 (3)	C15—H15B	0.9700
N2—C12	1.334 (3)	C15—H15C	0.9700
N2—C8	1.342 (3)	C16—C17	1.528 (3)
C1—C2	1.386 (3)	C16—H16B	0.9700
C1—C6	1.526 (3)	C16—H16C	0.9700
C2—C3	1.394 (3)	C17—C18	1.521 (3)
C2—H2A	0.9300	C17—H17A	0.9700
C3—C4	1.397 (3)	C17—H17B	0.9700
C3—H3A	0.9300	C18—H18A	0.9700
C4—C5	1.388 (3)	C18—H18B	0.9700
C4—H4A	0.9300	O1W—H1WA	0.7580
C5—C7	1.526 (3)	O1W—H1WB	0.7927
C8—C9	1.384 (3)	O2W—H2WA	0.8246
C8—C13	1.523 (3)	O2W—H2WB	0.8183
C9—C10	1.393 (3)		
			
N1—Cd1—N2	170.37 (6)	C11—C10—H10A	120.4
N1—Cd1—O1	71.35 (6)	C9—C10—H10A	120.4
N2—Cd1—O1	103.25 (6)	C12—C11—C10	118.90 (19)
N1—Cd1—O5	116.42 (6)	C12—C11—H11A	120.5
N2—Cd1—O5	70.76 (5)	C10—C11—H11A	120.5
O1—Cd1—O5	89.41 (6)	N2—C12—C11	121.14 (19)
N1—Cd1—O3	70.59 (6)	N2—C12—C14	115.98 (18)
N2—Cd1—O3	115.25 (5)	C11—C12—C14	122.87 (18)
O1—Cd1—O3	141.50 (5)	O6—C13—O5	125.73 (19)
O5—Cd1—O3	102.30 (5)	O6—C13—C8	118.25 (18)
N1—Cd1—O7	102.18 (6)	O5—C13—C8	116.01 (17)
N2—Cd1—O7	70.31 (5)	O8—C14—O7	126.69 (19)
O1—Cd1—O7	98.60 (5)	O8—C14—C12	116.93 (18)
O5—Cd1—O7	141.06 (5)	O7—C14—C12	116.37 (18)
O3—Cd1—O7	94.81 (5)	C15—N3—H3NA	110.6
C6—O1—Cd1	118.20 (13)	C15—N3—H3NB	111.2
C7—O3—Cd1	118.05 (12)	H3NA—N3—H3NB	108.0
C13—O5—Cd1	118.57 (13)	C15—N3—H3NC	105.3
C14—O7—Cd1	117.15 (13)	H3NA—N3—H3NC	109.7
C5—N1—C1	121.14 (18)	H3NB—N3—H3NC	111.9
C5—N1—Cd1	120.09 (14)	C18—N4—H4NA	108.1
C1—N1—Cd1	118.76 (13)	C18—N4—H4NB	107.6
C12—N2—C8	120.84 (18)	H4NA—N4—H4NB	111.3
C12—N2—Cd1	119.97 (13)	C18—N4—H4NC	107.8
C8—N2—Cd1	119.19 (13)	H4NA—N4—H4NC	108.9
N1—C1—C2	121.36 (19)	H4NB—N4—H4NC	112.9
N1—C1—C6	114.67 (18)	N3—C15—C16	112.65 (17)
C2—C1—C6	123.87 (18)	N3—C15—H15B	109.1
C1—C2—C3	118.22 (19)	C16—C15—H15B	109.1
C1—C2—H2A	120.9	N3—C15—H15C	109.1
C3—C2—H2A	120.9	C16—C15—H15C	109.1
C2—C3—C4	119.8 (2)	H15B—C15—H15C	107.8
C2—C3—H3A	120.1	C15—C16—C17	112.01 (17)
C4—C3—H3A	120.1	C15—C16—H16B	109.2
C5—C4—C3	118.46 (19)	C17—C16—H16B	109.2
C5—C4—H4A	120.8	C15—C16—H16C	109.2
C3—C4—H4A	120.8	C17—C16—H16C	109.2
N1—C5—C4	120.96 (19)	H16B—C16—H16C	107.9
N1—C5—C7	114.49 (18)	C18—C17—C16	112.08 (17)
C4—C5—C7	124.55 (18)	C18—C17—H17A	109.2
O2—C6—O1	126.3 (2)	C16—C17—H17A	109.2
O2—C6—C1	116.86 (18)	C18—C17—H17B	109.2
O1—C6—C1	116.76 (18)	C16—C17—H17B	109.2
O4—C7—O3	125.23 (19)	H17A—C17—H17B	107.9
O4—C7—C5	118.00 (18)	N4—C18—C17	110.61 (17)
O3—C7—C5	116.76 (17)	N4—C18—H18A	109.5
N2—C8—C9	120.98 (18)	C17—C18—H18A	109.5
N2—C8—C13	115.40 (17)	N4—C18—H18B	109.5
C9—C8—C13	123.63 (18)	C17—C18—H18B	109.5
C8—C9—C10	118.89 (19)	H18A—C18—H18B	108.1
C8—C9—H9A	120.6	H1WA—O1W—H1WB	108.6
C10—C9—H9A	120.6	H2WA—O2W—H2WB	105.3
C11—C10—C9	119.24 (19)		
			
N1—Cd1—O1—C6	−3.07 (15)	C1—N1—C5—C7	−178.33 (17)
N2—Cd1—O1—C6	168.63 (15)	Cd1—N1—C5—C7	1.7 (2)
O5—Cd1—O1—C6	−121.31 (16)	C3—C4—C5—N1	0.5 (3)
O3—Cd1—O1—C6	−12.1 (2)	C3—C4—C5—C7	179.94 (19)
O7—Cd1—O1—C6	96.96 (16)	Cd1—O1—C6—O2	178.27 (17)
N1—Cd1—O3—C7	0.66 (14)	Cd1—O1—C6—C1	1.3 (2)
N2—Cd1—O3—C7	−171.05 (14)	N1—C1—C6—O2	−174.47 (18)
O1—Cd1—O3—C7	9.71 (18)	C2—C1—C6—O2	2.0 (3)
O5—Cd1—O3—C7	114.61 (14)	N1—C1—C6—O1	2.8 (3)
O7—Cd1—O3—C7	−100.58 (14)	C2—C1—C6—O1	179.3 (2)
N1—Cd1—O5—C13	−170.64 (14)	Cd1—O3—C7—O4	178.78 (16)
N2—Cd1—O5—C13	2.38 (14)	Cd1—O3—C7—C5	0.0 (2)
O1—Cd1—O5—C13	−101.88 (14)	N1—C5—C7—O4	−179.97 (18)
O3—Cd1—O5—C13	115.11 (14)	C4—C5—C7—O4	0.6 (3)
O7—Cd1—O5—C13	1.14 (18)	N1—C5—C7—O3	−1.1 (3)
N1—Cd1—O7—C14	177.80 (14)	C4—C5—C7—O3	179.49 (19)
N2—Cd1—O7—C14	4.07 (14)	C12—N2—C8—C9	−0.4 (3)
O1—Cd1—O7—C14	105.15 (14)	Cd1—N2—C8—C9	179.68 (14)
O5—Cd1—O7—C14	5.33 (18)	C12—N2—C8—C13	−179.87 (17)
O3—Cd1—O7—C14	−111.05 (14)	Cd1—N2—C8—C13	0.2 (2)
O1—Cd1—N1—C5	−175.36 (16)	N2—C8—C9—C10	0.9 (3)
O5—Cd1—N1—C5	−95.72 (15)	C13—C8—C9—C10	−179.67 (18)
O3—Cd1—N1—C5	−1.29 (14)	C8—C9—C10—C11	−0.7 (3)
O7—Cd1—N1—C5	89.55 (15)	C9—C10—C11—C12	0.1 (3)
O1—Cd1—N1—C1	4.68 (14)	C8—N2—C12—C11	−0.3 (3)
O5—Cd1—N1—C1	84.32 (15)	Cd1—N2—C12—C11	179.66 (14)
O3—Cd1—N1—C1	178.74 (16)	C8—N2—C12—C14	−179.80 (17)
O7—Cd1—N1—C1	−90.41 (15)	Cd1—N2—C12—C14	0.1 (2)
O1—Cd1—N2—C12	−96.51 (15)	C10—C11—C12—N2	0.4 (3)
O5—Cd1—N2—C12	178.85 (16)	C10—C11—C12—C14	179.90 (18)
O3—Cd1—N2—C12	83.98 (15)	Cd1—O5—C13—O6	176.78 (16)
O7—Cd1—N2—C12	−1.98 (14)	Cd1—O5—C13—C8	−3.1 (2)
O1—Cd1—N2—C8	83.43 (15)	N2—C8—C13—O6	−177.92 (17)
O5—Cd1—N2—C8	−1.21 (13)	C9—C8—C13—O6	2.6 (3)
O3—Cd1—N2—C8	−96.08 (14)	N2—C8—C13—O5	1.9 (3)
O7—Cd1—N2—C8	177.96 (16)	C9—C8—C13—O5	−177.53 (19)
C5—N1—C1—C2	−2.2 (3)	Cd1—O7—C14—O8	175.41 (16)
Cd1—N1—C1—C2	177.80 (15)	Cd1—O7—C14—C12	−5.4 (2)
C5—N1—C1—C6	174.40 (18)	N2—C12—C14—O8	−177.06 (17)
Cd1—N1—C1—C6	−5.6 (2)	C11—C12—C14—O8	3.4 (3)
N1—C1—C2—C3	1.4 (3)	N2—C12—C14—O7	3.6 (3)
C6—C1—C2—C3	−174.79 (19)	C11—C12—C14—O7	−175.88 (19)
C1—C2—C3—C4	0.2 (3)	N3—C15—C16—C17	175.10 (17)
C2—C3—C4—C5	−1.2 (3)	C15—C16—C17—C18	61.0 (2)
C1—N1—C5—C4	1.1 (3)	C16—C17—C18—N4	174.37 (17)
Cd1—N1—C5—C4	−178.82 (15)		

### Hydrogen-bond geometry (Å, °)

**Table:**

*D*—H···*A*	*D*—H	H···*A*	*D*···*A*	*D*—H···*A*
N3—H3NA···O2W	0.89	1.90	2.782 (2)	169
N3—H3NB···O7^i^	0.91	1.93	2.823 (2)	166
N3—H3NC···O4^ii^	0.90	1.93	2.784 (2)	159
N4—H4NA···O8^ii^	0.91	1.99	2.821 (3)	152
N4—H4NB···O6^iii^	0.94	1.93	2.865 (2)	176
N4—H4NC···O1W^iv^	0.86	1.94	2.797 (2)	175
O1W—H1WA···O5^iv^	0.76	1.92	2.678 (2)	173
O1W—H1WB···O2	0.79	1.87	2.653 (2)	172
O2W—H2WA···O4^v^	0.82	1.99	2.809 (2)	175
O2W—H2WB···O1W	0.82	1.97	2.781 (2)	170

Symmetry codes: (i) *x*−1, *y*, *z*; (ii) *x*−1, *y*, *z*+1; (iii) *x*, −*y*+3/2, *z*+3/2; (iv) *x*, *y*, *z*+1; (v) −*x*+1, −*y*+2, −*z*.
